# Supplementary material for: The middle lipin domain adopts a membrane-binding dimeric protein fold
Source: Nat Commun. 2021 Aug 5;12:4718. doi: 10.1038/s41467-021-24929-5 (PMC8342540; doi:10.1038/s41467-021-24929-5)
Supplement: Supplementary file 1 — Supplementary information [file 41467_2021_24929_MOESM1_ESM.pdf]

Supplementary Information

Gu et al

The middle lipin domain adopts a membrane-binding dimeric protein fold

Supplementary Table 1. Primer List

Construct [subcloning] or Gene (RT-PCR)	Forward Primer (5'-3')	Reverse Primer (5'-3')
Lipin1α 1-891 in YM-Bac3	Lipin1-SfoI-1F GGCGACGGCGCCATGAAT TACGTGGGGCAGCTGGC	Lipin1-891-NotI-Stop-R GGCGACGCGGCCGCTCAAGC TGAGGCTGAATGCATGTCCTG
Lipin1α 1-891 Δ458-565 in YM-Bac3	Lipin1-Δ458-565-F AGCACCTCCGACAAAGAGGA AAGCAAGCCTGAACAGTGC	Lipin1-Δ458-565-R TGCTTTCCTCTTTGTCGG AGGTGCTCTCCACACCAC
Lipin1 458-565 in pET28b	Lipin1-NdeI-458F GGCGACCATATGCTGAG GGACCTGCCATCCATCG	Lipin1-NotI-565R GGCGACGCGGCCGCCTAGATT GTGGCATTTCTTCCTCTCCAG
Lipin1 458-548 in pET28b	Lipin1-NdeI-458F GGCGACCATATGCTGAG GGACCTGCCATCCATCG	Lipin1-NotI-548R GGCGACGCGGCCGCCTAGGG CATCTTATCTCTCATGATGGAT
Lipin1 458-548 in ppSUMO	Lipin1-BamHI-458F GGCGACGGATCCCTGAG GGACCTGCCATCCATCG	Lipin1-NotI-548R GGCGACGCGGCCGCCTAGGG CATCTTATCTCTCATGATGGAT
Lipin2 459-549 in pET28b	Lipin2-NdeI-459F GGCGACCATATGATGAAT TATGTGGGCCAGCTGGCT	Lipin2-NotI-549R GGCGACGCGGCCGCCTATGG CATCTTGTCTTTAACCCAGGAC
Lipin1α 1-891 in pcDNA3.1	Lipin1-EcoRI-1F GGCGACGAATTCCCACCATGA ATTACGTGGGGCAGCTGGC	Lipin1-891-NotI-R GGCGACGCGGCCGCAGCTG AGGCTGAATGCATGTCCTG
Lipin1α 1-891 Δ458-565 in pcDNA3.1	Lipin1-Δ458-565-F AGCACCTCCGACAAAGAGGA AAGCAAGCCTGAACAGTGC	Lipin1-Δ458-565-R TGCTTTCCTCTTTGTCGG AGGTGCTCTCCACACCAC
Lipin1α 1-891 Δ458-548 in pcDNA3.1	Lipin1-Δ458-548-F AGCACCTCCGACAAAAAGGG AGGAAGATGGTGGTTTTCCT	Lipin1-Δ458-548-R TCCTCCCTTTTTGTCGGA GGTGCTCTCCACACCAC
Lipin1α 1-891-mEGFP and Lipin1α 1-891 Δ458-565-mEGFP in pcDNA3.1	mEGFP-Age-F CGTACCGGTATGGTG AGCAAGGGCGAG	mEGFP-PmeStop-R AGCTTTGTTTAAACTCACTT GTACAGCTCATCCATGC
Pparg	AACTCTGGGAGATTCTCCTGTTGA	TGGTAATTTCTTGTGAAGTGCTCATA
Cebpa	GAACAGCAACGAGTAACCGGGTA	GCCATGGCCTTGACCAAGGAG
Fabp4	AACCTGGAAGCTTGTCTCCA	CACGCCCAGTTTGAAGGAAA
Adipoq	TGTTCCTCTTAATCCTGCCCA	CCAACCTGCACAAGTTCCCTT
Acaca	GCCTCTTCCTGACAAACGAG	TGACTGCCGAAACATCTCTG
Fasn	GTTGGCCCAGAACTCCTGTA	GTCGTCTGCCTCCAGAGC
Dgat1	CTGAATTGGTGTGTGGTGATG	AGGGGTCCTTCAGAAACAGAG
Tbp	ACCCTTCACCAATGACTCCTATG	ATGATGACTGCAGCAAATCGC

Supplementary Table 2. HDX Data Summary

Data Set	lipin-1 order/disorder	lipin-1	lipin-1 + membrane	M-Lip domain	M-Lip domain + membrane
HDX reaction details	%D2O=84.8% pH(read)= 7.5 Temp= 4°C	%D2O=80.1% pH(read)= 7.5 Temp= 18°C	%D2O=80.1% pH(read)= 7.5 Temp= 18°C	%D2O=85% pH(read)= 7.5 Temp= 18°C	%D2O=85% pH(read)= 7.5 Temp= 18°C
HDX time course	3s	3s, 30s, 300s, 3000s	3s, 30s, 300s, 3000s	3s, 30s, 300s, 3000s	3s, 30s, 300s, 3000s
HDX controls	Back exchange correction	N/A	N/A	N/A	N/A
Back-exchange	Average back-exchange = 32.2% (IQR=22.5%-40%)	Corrected based on %D2O	Corrected based on %D2O	Corrected based on %D2O	Corrected based on %D2O
Number of peptides	189	179	179	82	82
Sequence coverage	88.8%	90.6%	90.6%	99.2%	99.2%
Average peptide length / Redundancy	Length = 15.8 Redundancy = 3.1	Length = 15.9 Redundancy = 3.0	Length = 15.9 Redundancy = 3.0	Length = 13.2 Redundancy = 8.4	Length = 13.2 Redundancy = 8.4
Replicates	3	3	3	3	3
Repeatability	Average StDev = 0.6%	Average StDev = 0.8%	Average StDev = 0.7%	Average StDev = 0.5%	Average StDev = 0.5%
Significant differences in HDX	N/A	>4% and >0.4 Da and unpaired t-test <0.01	>4% and >0.4 Da and unpaired t-test <0.01	>4% and >0.4 Da and unpaired t-test <0.01	>4% and >0.4 Da and unpaired t-test <0.01

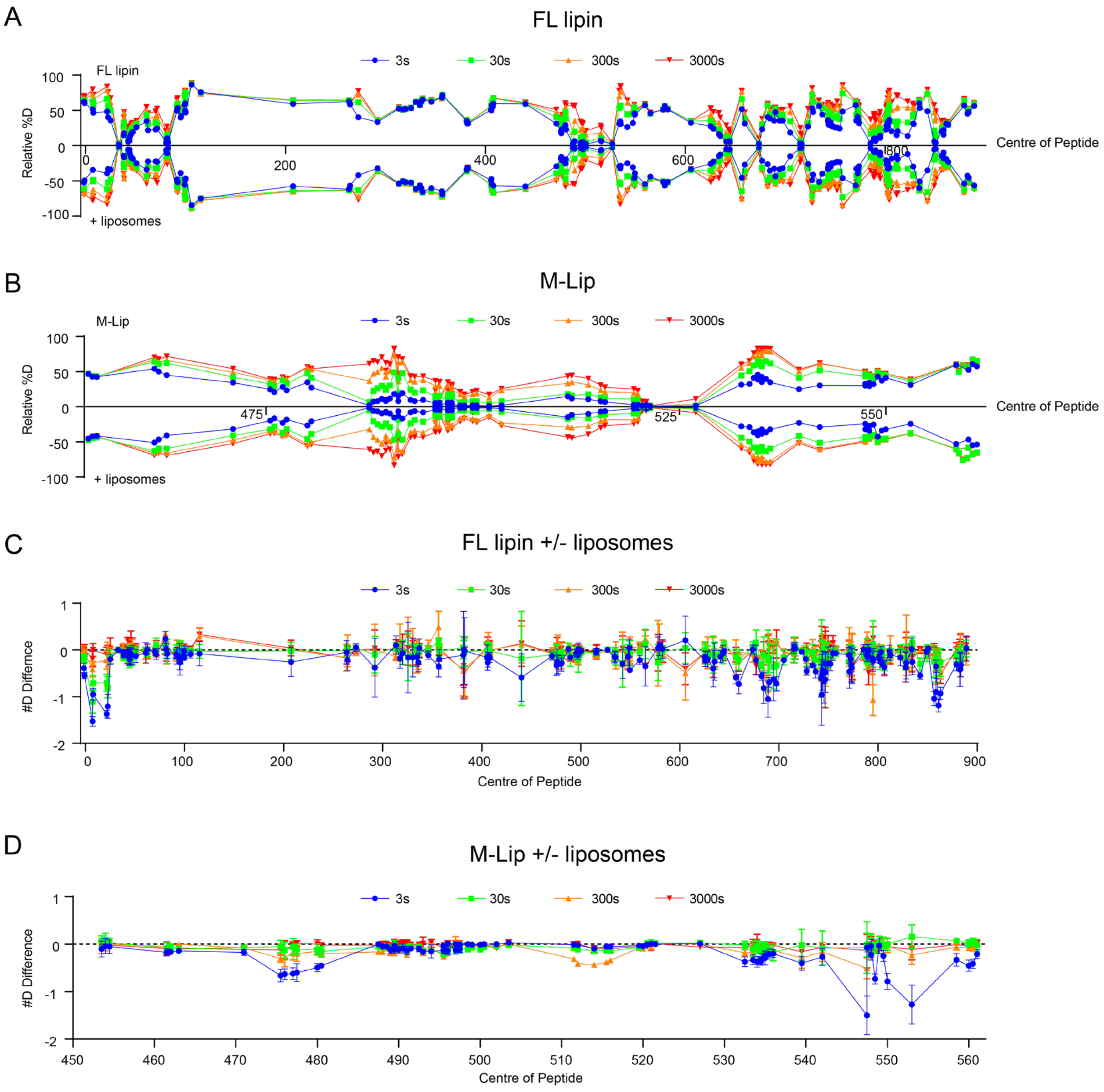

Supplementary Figure 1. Butterfly and differential plots for HDX comparing full-length lipin and the M-Lip domain with liposomes.

(a) Butterfly plot for deuterium incorporation of full-length lipin in the presence and absence of liposomes. For all graphs the colors indicated the different timepoints, with every peptide being represented by a single point graphed according to the central residue. **(b)** Butterfly plot for deuterium incorporation of the M-Lip domain in the presence and absence of liposomes. **(c)** Differences in the # deuterium incorporation between full-length lipin alone and full-length lipin with liposomes. **(d)** Differences in the # deuterium incorporation between the M-Lip domain alone and the M-Lip domain with liposomes. Data are presented as mean values +/- SD. n=3 independent experiments. Source data are provided as a Source Data file.

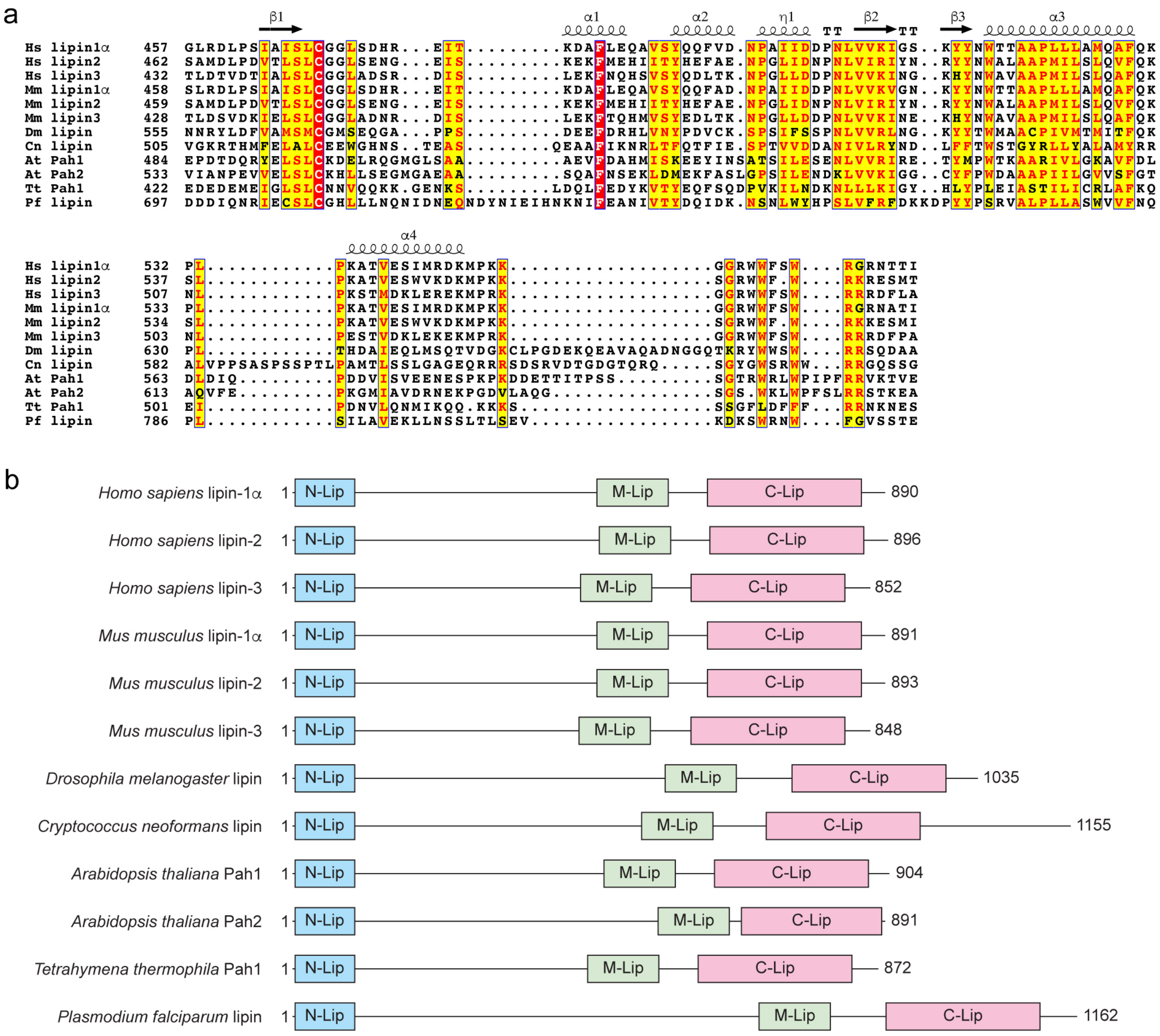

Supplementary Figure 2. Evolutionary conservation of the M-Lip domain

(a) Sequence alignment of the M-Lip domain from evolutionarily distant organisms. Identical residues are shaded red and residues with positive homology are shaded yellow. Secondary structure elements for the mouse lipin 1 M-Lip domain are indicated above, “TT” = turn.

(b) Domain architecture of lipin/Pahs that contain the M-Lip domain, drawn to scale. Other conserved motifs, e.g. nuclear localization signals, predicted amphipathic helices, and the conserved Trp motif are omitted for clarity.

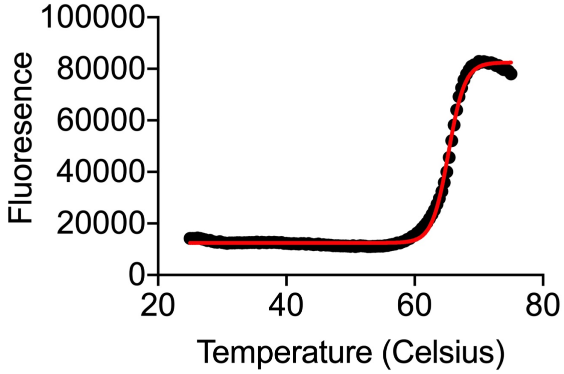

Supplementary Figure 3. Thermal shift assay.

The thermal stability of the mouse lipin-1 M-Lip^{xtal} domain was assessed by differential scanning fluorimetry using Sypro Orange (Sigma Aldrich). Individual data points are shown as black circles. A Boltzmann sigmoidal fit (red line) was used to determine the melting temperature. The data indicate a melting temperature of 65°C.

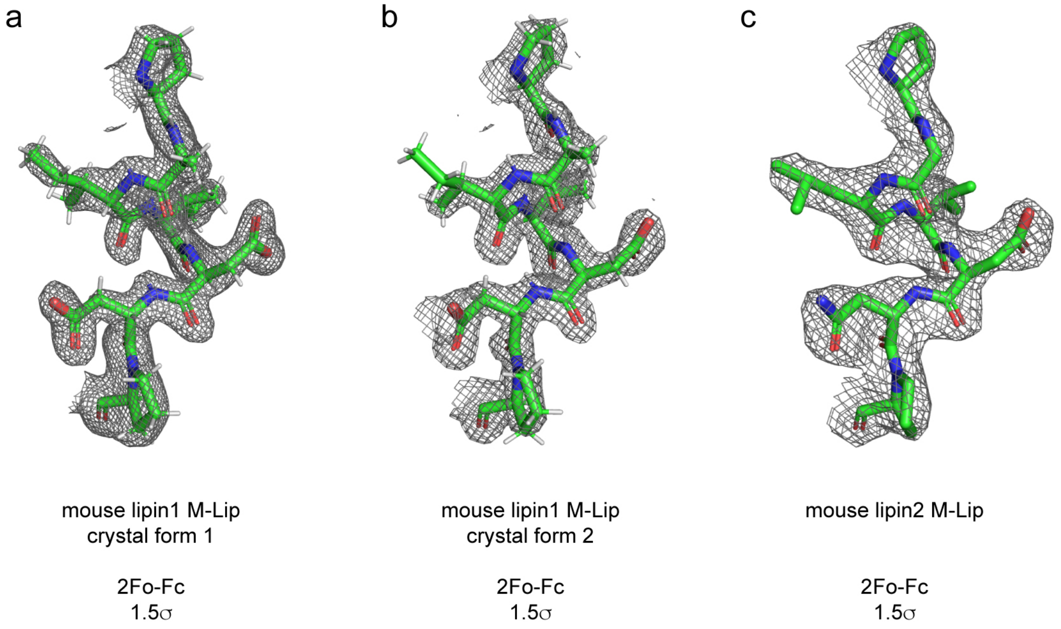

Supplementary Figure 4. Electron density maps.

2Fo-Fc electron density maps contoured at 1.5 sigma of **(a)** the mouse lipin 1 M-Lip^{xtal} domain from crystal form 1, **(b)** the mouse lipin 1 M-Lip^{xtal} domain from crystal form 2, and **(c)** the mouse lipin 2 M-Lip^{xtal} domain.

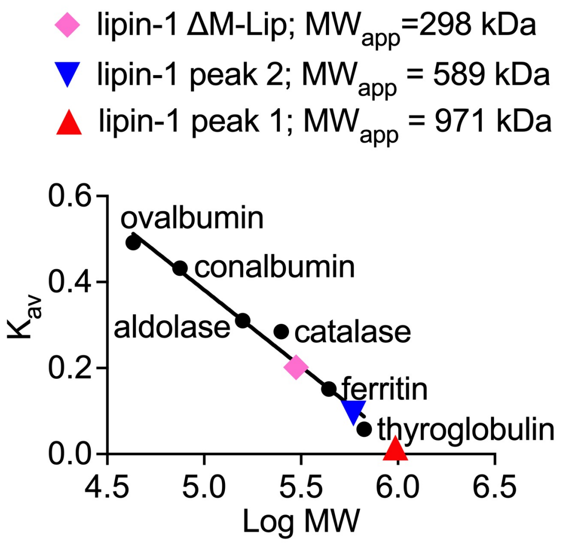

Supplementary Figure 5. Size exclusion chromatography calibration curve with proteins of known molecular weight.

Deletion of the M-Lip results in an apparent molecular weight approximately half the size of wild type lipin (298 kDa vs. 589 kDa). The long, disordered linker between the N-Lip and M-Lip most likely increase the apparent molecular weight, as this region is not globular. The 1st peak of wild type lipin has an apparent molecular weight of 971 kDa, but is close to the void volume, making the calculation less accurate. Source data are provided as a Source Data file.

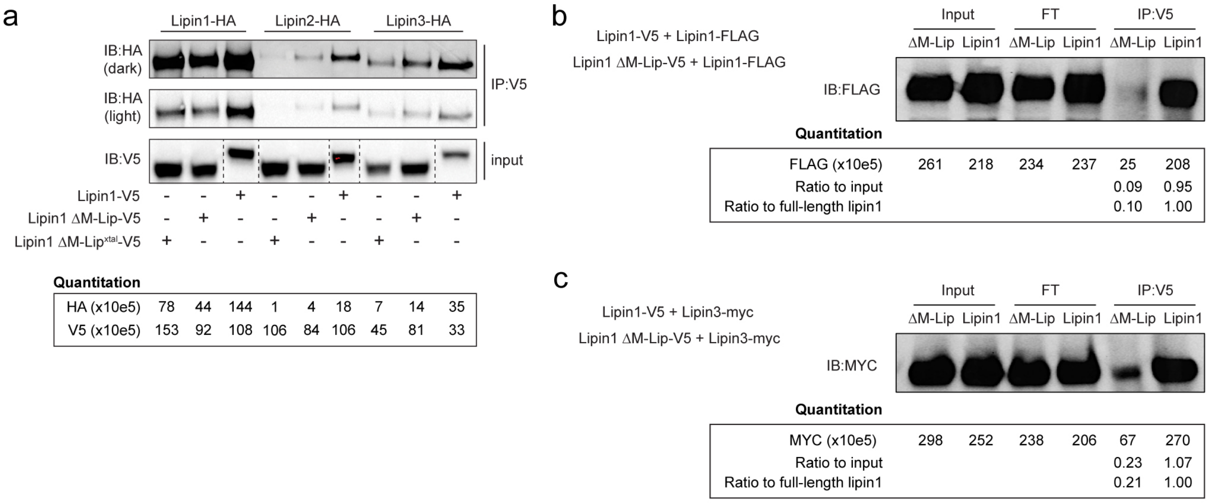

Supplementary Figure 6. Deletion of the M-Lip reduces lipin homo- and hetero-oligomerization.

(a) Co-immunoprecipitation of HA-tagged lipins with V5-tagged lipin 1 constructs shown in Figure 2h with quantitation below by densitometry. Within a blot, all lanes have identical protein loads. The Input blot represents 10% of the total protein used for immunoprecipitation reactions. Densitometric levels can only be compared within blots, and not across blots, as they are with different antibodies. n=1 independent experiment.

(b) Co-immunoprecipitation of V5-tagged lipin-1 or ΔM-Lip constructs with FLAG-lipin 1 in Hepa1-6 cells. Quantitation by densitometry was used to calculate the relative amounts of full-length or ΔM-Lip lipin 1 that immunoprecipitated with lipin 1. FT, flow-through from the immunoprecipitate reaction. The 'input' lanes contain 10% of total protein from the transfected cells; the remainder was used for immunoprecipitation. FT, flow-through from immunoprecipitation. n=1 independent experiment.

(c) Co-immunoprecipitation of V5-tagged lipin-1 and ΔM-Lip constructs with Myc-tagged lipin 3 in Hepa1-6 cells as described in (b). Quantitation by densitometry was used to calculate the relative amounts of full-length or ΔM-Lip lipin 1 that immunoprecipitated with lipin 3. n=1 independent experiment.

Source data are provided as a Source Data file.

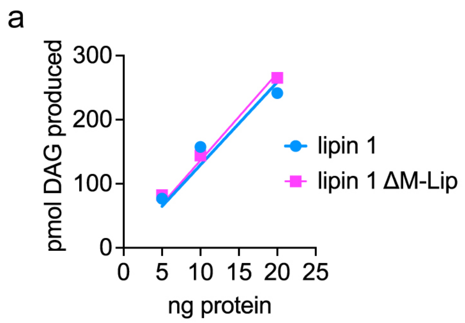

Supplementary Figure 7. PAP assay linearity.

(a) PAP activity assays were linear in respect to protein concentration and **(b)** to time.

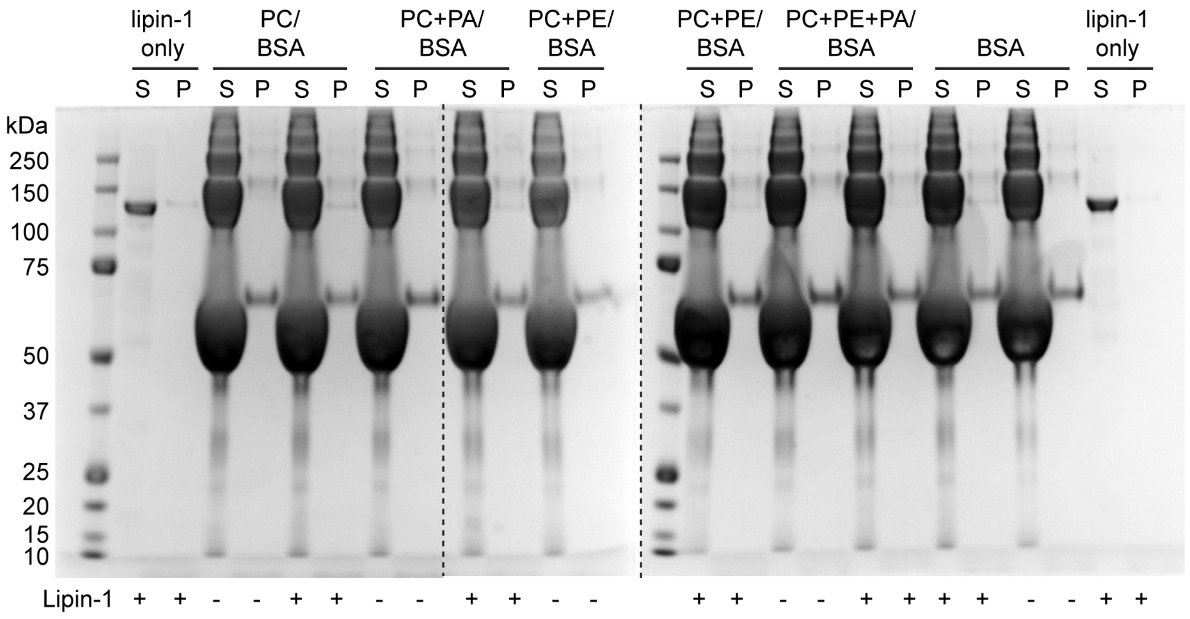

Supplementary Figure 8. Sedimentation assays of lipin 1 with BSA/lipid complexes. Lipids complexed with BSA do not induce aggregation of lipin 1 and subsequent non-specific pelleting after ultracentrifugation. A small fraction of lipin 1 pellets after ultracentrifugation, and this is not affected by BSA or BSA complexed to lipids. The total phospholipid concentration was equal to 1mM and is identical to the phospholipid concentration in corresponding liposome sedimentation assays. BSA, fatty acid free BSA; PC, phosphatidylcholine; PE, phosphatidylethanolamine; PA, phosphatidic acid. The presence or absence of lipin 1 is indicated by + and – symbols respectively. n=1 independent experiment.

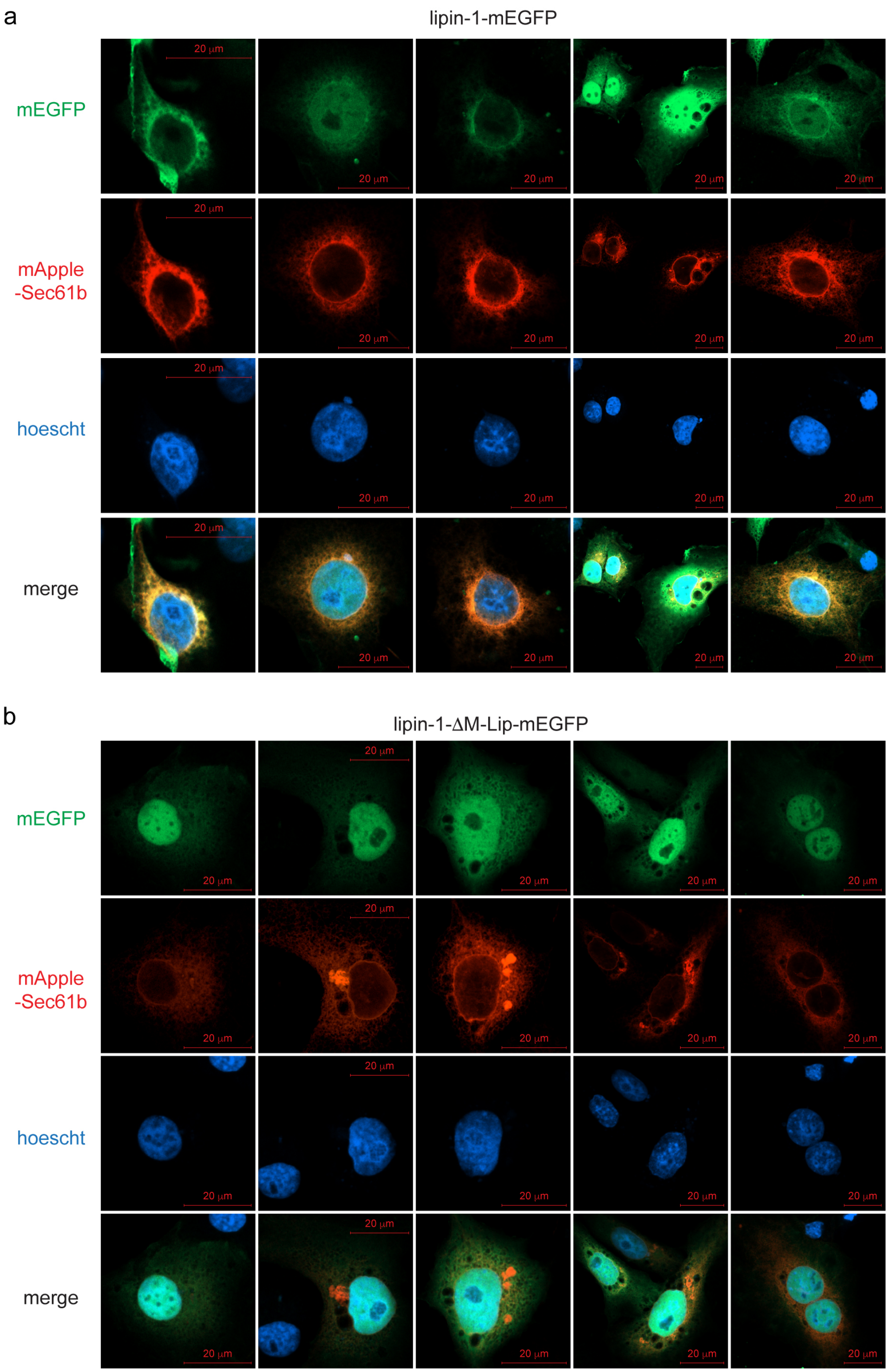

Supplementary Figure 9. Lipin subcellular localization. Confocal microscopy images of Cos-7 cells transiently transfected with monomeric enhanced GFP (mEGFP) fusions of either **(a)** lipin-1 or **(b)** lipin-1 ΔM-Lip (green) and the ER marker mApple-Sec61b (red). Hoechst stain (blue), nucleus. Scale bar: 20 μm. n=5 with independent images shown in Fig. 3g.

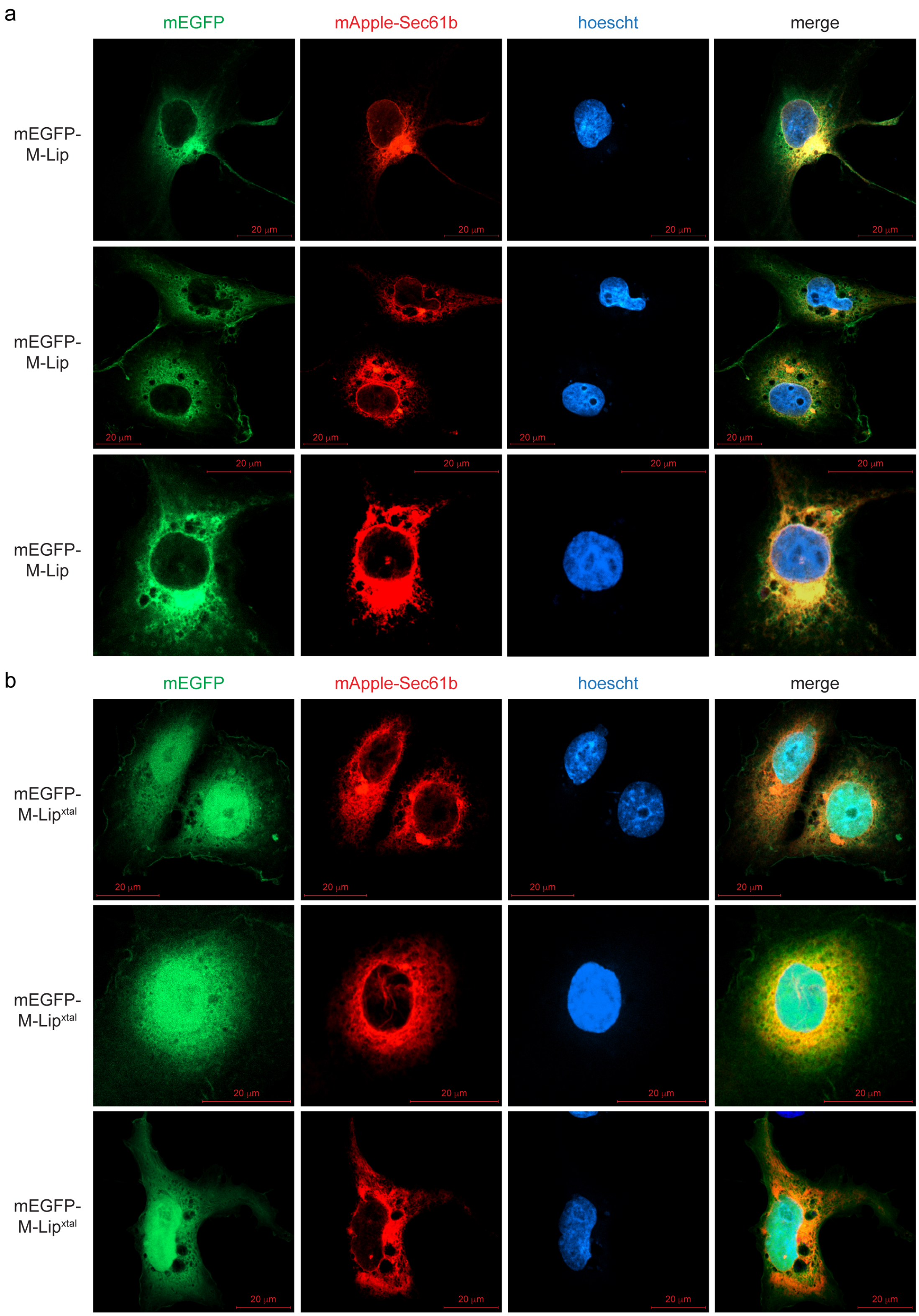

Supplementary Figure 10. M-Lip subcellular localization. Confocal microscopy images of Cos-7 cells transiently transfected with monomeric enhanced GFP (mEGFP) fusions of either the **(a)** M-Lip or **(b)** M-Lip^{xtal} domains (green) and the ER marker mApple-Sec61b (red). Hoechst stain (blue), nucleus. Scale bar: 20 μm. n=3 with independent images shown in Fig. 4d.

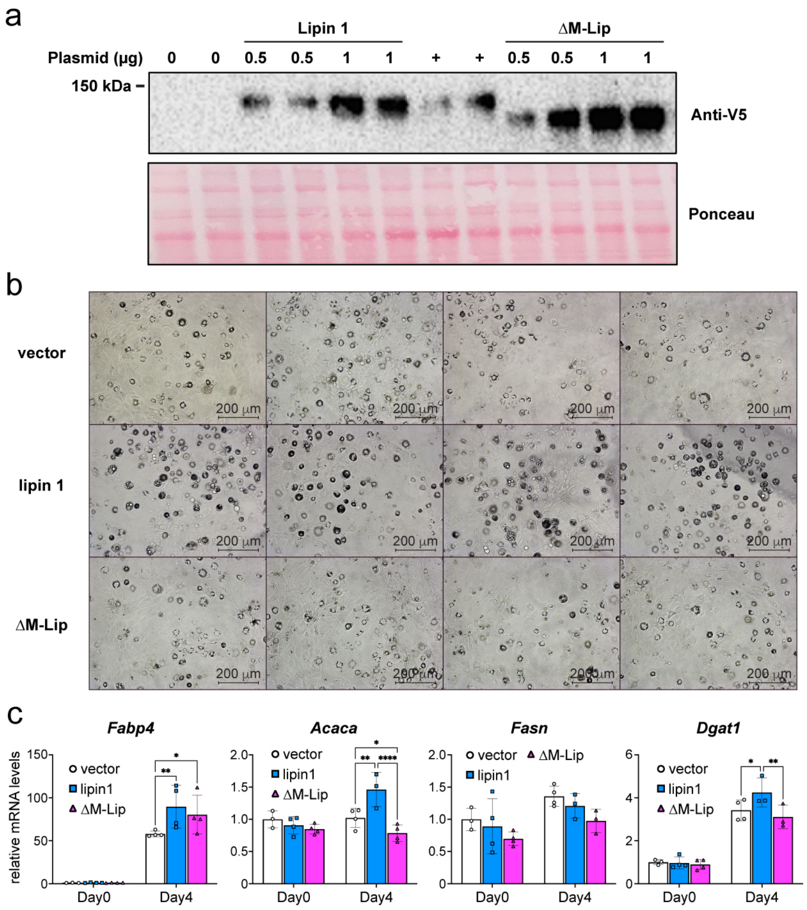

Supplementary Figure 11. Additional data regarding role of M-Lip in lipin 1 enhancement of adipogenesis.

(a) Similar protein levels of wild-type lipin 1 and ΔM-Lip protein expression in 3T3-L1 transfection experiments. For experiments shown, 0.5 µg of each construct was used for transfections. +, recombinant lipin 1 produced in HEK293 cells as control for antibody detection. Total protein loads shown with Ponceau-stained blot. n=1 independent experiment.

(b) Lipid accumulation at intermediate stage (day 5) of 3T3-L1 differentiation in an independent experiment from that shown in Fig. 5b. Bright field illumination shows lipid accumulation as opaque areas within cells (100x). Scale bar: 200 μm. n=4 with independent replicate experiment shown in Fig. 5b.

(c) Gene expression from an independent experiment to that in Fig. 5c showing expression of genes involved in fatty acid synthesis (*Acaca*, *Fasn*) and triglyceride synthesis (*Dgat1*). *Fabp4*, fatty acid binding protein 4; *Acaca*, acetyl CoA-carboxylase; *Fasn*, fatty acid synthase; *Dgat1*, diacylglycerol acyltransferase 1. Gene expression was analyzed by 2-way ANOVA. *, $p<0.05$; **, $p<0.01$; ***$p<0.001$. Data are presented as mean values +/- SD. n=4 biologically independent experiments.

Source data are provided as a Source Data file.
