# Supplementary material for: The middle lipin domain adopts a membrane-binding dimeric protein fold
Source: Nat Commun. 2021 Aug 5;12:4718. doi: 10.1038/s41467-021-24929-5 (PMC8342540; doi:10.1038/s41467-021-24929-5)
Supplement: Supplementary file 3 — Reporting Summary [file 41467_2021_24929_MOESM3_ESM.pdf]

nature portfolio

Corresponding author(s): Michael Airola and Karen Reue

Last updated by author(s): Jul 1, 2021

Reporting Summary

Nature Portfolio wishes to improve the reproducibility of the work that we publish. This form provides structure for consistency and transparency in reporting. For further information on Nature Portfolio policies, see our Editorial Policies and the Editorial Policy Checklist.

Statistics

For all statistical analyses, confirm that the following items are present in the figure legend, table legend, main text, or Methods section.

n/a	Confirmed	
☐	☒	The exact sample size (n) for each experimental group/condition, given as a discrete number and unit of measurement
☐	☒	A statement on whether measurements were taken from distinct samples or whether the same sample was measured repeatedly
☐	☒	The statistical test(s) used AND whether they are one- or two-sided<br>*Only common tests should be described solely by name; describe more complex techniques in the Methods section.*
☒	☐	A description of all covariates tested
☒	☐	A description of any assumptions or corrections, such as tests of normality and adjustment for multiple comparisons
☐	☒	A full description of the statistical parameters including central tendency (e.g. means) or other basic estimates (e.g. regression coefficient) AND variation (e.g. standard deviation) or associated estimates of uncertainty (e.g. confidence intervals)
☐	☒	For null hypothesis testing, the test statistic (e.g. F, t, r) with confidence intervals, effect sizes, degrees of freedom and P value noted<br>*Give P values as exact values whenever suitable.*
☒	☐	For Bayesian analysis, information on the choice of priors and Markov chain Monte Carlo settings
☒	☐	For hierarchical and complex designs, identification of the appropriate level for tests and full reporting of outcomes
☒	☐	Estimates of effect sizes (e.g. Cohen's d, Pearson's r), indicating how they were calculated

Our web collection on statistics for biologists contains articles on many of the points above.

Software and code

Policy information about availability of computer code

Data collection	no software was used
Data analysis	Coot 0.8.9.1<br>Phenix 1.15rc1-3423<br>CCP4 7.0.000<br>GraphPad Prism 8.1<br>HDExaminer (Sierra Analytics) version 2.5.1<br>PEAKS Client 7.0 (BSI)<br>Image J (153)

For manuscripts utilizing custom algorithms or software that are central to the research but not yet described in published literature, software must be made available to editors and reviewers. We strongly encourage code deposition in a community repository (e.g. GitHub). See the Nature Portfolio guidelines for submitting code & software for further information.

Data

Policy information about availability of data

All manuscripts must include a data availability statement. This statement should provide the following information, where applicable:

- Accession codes, unique identifiers, or web links for publicly available datasets
- A description of any restrictions on data availability
- For clinical datasets or third party data, please ensure that the statement adheres to our policy

The coordinates and structure factors in this study have been deposited in the Protein Data Bank under accession codes 7KIH [https://doi.org/10.2210/pdb7KIH/

pdb], 7KIL [https://doi.org/10.2210/pdb7KIL/pdb], and 7KIQ [https://doi.org/10.2210/pdb7KIQ/pdb]. The mass spectrometry proteomics data in this study have been deposited in the ProteomeXchange Consortium via the PRIDE partner repository44 with the dataset identifier PXD022172 [http://proteomecentral.proteomexchange.org/cgi/GetDataset?ID=PXD022172]. Source data are provided with this paper.

Field-specific reporting

Please select the one below that is the best fit for your research. If you are not sure, read the appropriate sections before making your selection.

- [x] Life sciences
- [ ] Behavioural & social sciences
- [ ] Ecological, evolutionary & environmental sciences

For a reference copy of the document with all sections, see nature.com/documents/nr-reporting-summary-flat.pdf

Life sciences study design

All studies must disclose on these points even when the disclosure is negative.

Sample size	Sample sizes are given in the manuscript. All assays were repeated at least three times, which is sufficient to derive error bars, p values, and statistical significance.
Data exclusions	No data was excluded from the analyses.
Replication	All experimental findings were confirmed with at least three independent replicates as detailed in Methods or Figure Legends. All attempts at replication were successful.
Randomization	All assays were conducted with wild type lipin in order to test the effect of deletion of the M-Lip domain. No randomization is needed for these assays. For structure determination, 5% of the reflections were randomly included in the Rfree calculations, as per standard X-ray crystallography refinement procedures.
Blinding	No blinding was performed as the bias from prior knowledge of samples in case of biochemical, blotting, and imagining experiments described in the study is considered limited. All samples including controls were analyzed in exactly the same manner.

Reporting for specific materials, systems and methods

We require information from authors about some types of materials, experimental systems and methods used in many studies. Here, indicate whether each material, system or method listed is relevant to your study. If you are not sure if a list item applies to your research, read the appropriate section before selecting a response.

Materials & experimental systems

n/a	Involved in the study	
[ ]	[x]	Antibodies
[ ]	[x]	Eukaryotic cell lines
[x]	[ ]	Palaeontology and archaeology
[x]	[ ]	Animals and other organisms
[x]	[ ]	Human research participants
[x]	[ ]	Clinical data
[x]	[ ]	Dual use research of concern

Methods

n/a	Involved in the study	
[x]	[ ]	ChIP-seq
[x]	[ ]	Flow cytometry
[x]	[ ]	MRI-based neuroimaging

Antibodies

Antibodies used	Commerical antibodies against V5 (R960-25, ThermoFisher Scientific, Waltham, MA), HA (3724, Cell Signaling, Danvers, MA), FLAG (PA1-984B, Invitrogen, Carlsbad, CA), and Myc (2278, Cell Signaling, Danvers, MA) epitopes. The dilution used for each antibody is specified in the methods of the manuscript.
Validation	All antibodies were validated by suppliers for the use at least in immunoblotting and immunoprecipitation, and in our laboratory with appropriate positive and negative controls (recombinant proteins containing or lacking these epitopes, respectively). The commercial anti-V5 antibody was validated by ThermoFisher (https://www.thermofisher.com/antibody/product/V5-Tag-Antibody-Monoclonal/R960-25). The commercial anti-HA antibody was validated by Cell Signaling (https://www.cellsignal.com/products/primary-antibodies/ha-tag-c29f4-rabbit-mab/3724). The commercial anti-FLAG antibody was validated by ThermoFisher (https://www.thermofisher.com/antibody/product/DYKDDDDK-Tag-Antibody-Polyclonal/PA1-984B). The commercial anti-Myc antibody was validated by Cell Signaling (https://www.cellsignal.com/products/primary-antibodies/myc-tag-71d10-rabbit-mab/2278).

Eukaryotic cell lines

Policy information about cell lines

Cell line source(s)	Human embryonic kidney 293 cells (HEK293, American Type Culture Collection #CRL-1573). Mouse 3T3-L1 preadipocytes were purchased from Zen-Bio (#SP-L1-F). Mouse Hepa1-6 cells were purchased from ATCC (CRL-1830). Cos-7 cells were purchased from ATCC (#CRL-1651)
Authentication	Cells were not authenticated in our lab. HEK293, Hepa1-6, and Cos-7 cell lines were authenticated with morphology, karyotyping, and PCR based approaches by ATCC. 3T3-L1 cells were authenticated with morphology, karyotyping, and PCR based approaches by Zen-Bio. Cells were carefully handled and expanded right after arrival. Early cell passage number 2-5 was used to ensure prevent genetic drift and mycoplasma contamination developed after too many passages. 3T3-L1 preadipocytes were used at passage numbers <4. COS-7 cells were maintained in DMEM containing 10% fetal bovine serum and penicillin/streptomycin and cultured at 37°C with 5% CO_2.
Mycoplasma contamination	Cell lines were not tested for mycoplasma contamination.
Commonly misidentified lines (See ICLAC register)	HEK293 cells were used to achieve high rates of transfection.
